# Prey preference follows phylogeny: evolutionary dietary patterns within the marine gastropod group Cladobranchia (Gastropoda: Heterobranchia: Nudibranchia)

**DOI:** 10.1186/s12862-017-1066-0

**Published:** 2017-10-26

**Authors:** Jessica A. Goodheart, Adam L. Bazinet, Ángel Valdés, Allen G. Collins, Michael P. Cummings

**Affiliations:** 1 Laboratory of Molecular Evolution, Center for Bioinformatics and Computational Biology, University of Maryland, College Park, MD 20742 USA; 2 NMFS, National Systematics Laboratory, National Museum of Natural History, Smithsonian Institution, MRC-153, PO Box 37012, Washington, DC 20013 USA; 3 Present address: National Biodefense Analysis and Countermeasures Center, 8300 Research Plaza, Fort Detrick, MD 21702 USA; 4 Department of Biological Sciences, California State Polytechnic University, 3801 W Temple Ave, Pomona, CA 91768 USA

**Keywords:** Mollusca, phylogenomics, nudibranchs, sea slugs, RNA-Seq, diet

## Abstract

**Background:**

The impact of predator-prey interactions on the evolution of many marine invertebrates is poorly understood. Since barriers to genetic exchange are less obvious in the marine realm than in terrestrial or freshwater systems, non-allopatric divergence may play a fundamental role in the generation of biodiversity. In this context, shifts between major prey types could constitute important factors explaining the biodiversity of marine taxa, particularly in groups with highly specialized diets. However, the scarcity of marine specialized consumers for which reliable phylogenies exist hampers attempts to test the role of trophic specialization in evolution. In this study, RNA-Seq data is used to produce a phylogeny of Cladobranchia, a group of marine invertebrates that feed on a diverse array of prey taxa but mostly specialize on cnidarians. The broad range of prey type preferences allegedly present in two major groups within Cladobranchia suggest that prey type shifts are relatively common over evolutionary timescales.

**Results:**

In the present study, we generated a well-supported phylogeny of the major lineages within Cladobranchia using RNA-Seq data, and used ancestral state reconstruction analyses to better understand the evolution of prey preference. These analyses answered several fundamental questions regarding the evolutionary relationships within Cladobranchia, including support for a clade of species from Arminidae as sister to Tritoniidae (which both preferentially prey on Octocorallia). Ancestral state reconstruction analyses supported a cladobranchian ancestor with a preference for Hydrozoa and show that the few transitions identified only occur from lineages that prey on Hydrozoa to those that feed on other types of prey.

**Conclusions:**

There is strong phylogenetic correlation with prey preference within Cladobranchia, suggesting that prey type specialization within this group has inertia. Shifts between different types of prey have occurred rarely throughout the evolution of Cladobranchia, indicating that this may not have been an important driver of the diversity within this group.

**Electronic supplementary material:**

The online version of this article (10.1186/s12862-017-1066-0) contains supplementary material, which is available to authorized users.

## Background

Predator-prey interactions are among the most fundamental processes in ecology and constitute the fabric of community structure and ecosystem function [1, 2]. However, the role of those interactions in evolution, and their impacts on biodiversity, are less well understood in marine systems [3, 4]. The most widely accepted hypothesis to explain the origin of biological diversity traces its origins to Mayr [5, 6], who proposed that the ranges of organisms are fragmented by the formation of physical barriers, resulting in isolation and divergence in allopatry. However, in the marine realm, where barriers to genetic exchange are less obvious than in terrestrial or freshwater systems [7], non-allopatric divergence and speciation may play a fundamental role in the generation of biodiversity (e.g., [8, 9]). In this context, shifts between major prey types (e.g., different cnidarian classes) could constitute important factors explaining the biodiversity of marine taxa, particularly in groups with highly specialized diets. The greatest obstacle to testing these ideas is the lack of well-supported phylogenies for groups of specialized consumers.

**Fig. 1:**
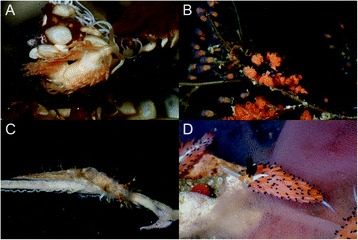
Select photographs of cladobranch taxa on their food source, including: (**a**) *Dondice parguerensis* on the scyphozoan jellyfish *Cassiopea* sp*.*, (**b**) *Doto chica* on the hydroid *Eudendrium* sp.; (**c**) *Tritonia hamnerorum* on the octocoral *Gorgonia ventalina*; and (**d**) *Favorinus tsuruganus* on an opisthobranch egg mass (Photo credits: Ángel Valdés)

In this study, we generate RNA-Seq data to test the role of prey preference shifting in the evolution of Cladobranchia (Mollusca: Gastropoda: Hererobranchia: Nudibranchia), a group of marine invertebrates with at least 1000 species [10]. Cladobranch sea slugs occupy various marine environments, from coastal reefs, where diversity is highest, to the deep sea, as well as highly specialized pelagic and neustonic niches [11–14]. Species of Cladobranchia are exclusively carnivorous, and exhibit diverse dietary specializations, preying on a variety of animal taxa, including bryozoans and crustaceans, eggs of fishes and molluscs, and cnidarians (Fig. 1) [15–17]. However, the vast majority of cladobranchs prey on species of the two most diverse clades within Cnidaria, Anthozoa (e.g., anemones, stony corals, and octocorals) and Hydrozoa (hydroids, siphonophores, and hydromedusae) [15, 18–20]. This preference for cnidarian prey is hypothesized to have facilitated the evolution of the ability to sequester cnidarian nematocysts in Cladobranchia [21], which is believed to have evolved only once within this group [16].

Based on recent classifications, two of the three main groups in Cladobranchia (Aeolidida and Dendronotida [22]; the third being Arminida, as defined in [23]) contain taxa that prey on animals distributed across the list given above. These classifications suggest that shifts in prey type preference are relatively common throughout Cladobranchia over evolutionary timescales. There also exist many well-documented cases where cladobranch species are tightly associated with specific prey types or species (e.g., [24–29]). In many of these cases the prey species might even be considered a host as defined by Coyne & Orr [93], due to similarities that many sea slugs share with herbivorous insects, including their small size relative to their hosts and the use of hosts for both food and shelter [3].

Two main hypotheses have emerged regarding the roles that dietary specialization and prey shifts have played in the evolution of heterobranch sea slugs (formerly called opisthobranchs). The first of these hypotheses suggests that increased speciation occurs due to species-specific prey switching in groups where specialization is prevalent [30]. This leads to clades consisting of many taxa that specialize on individual prey species. In many metazoan groups studied, mainly involving terrestrial symbiotic and parasitic systems [31–33], host shifting (shifting between prey species) has been implicated as a driver of diversification, with colonization of new hosts often leading to bursts of cladogenesis. Speciation of taxa by host or prey shifting may also be important in a handful of specialized marine consumers such as some bivalves [34], amphipods [35], barnacles [36], gobies [37], and gastropods [24]. We do not assess this hypothesis here, as it requires broad taxon sampling across Cladobranchia and solid evidence of dietary specialization, both of which are lacking in many cases.

The second hypothesis relating to dietary specialization is that major radiations within heterobranch sea slugs may be related to the evolution of particular morphological structures necessary for feeding on different types of prey [16, 17, 38], such as the distinct radular morphology present in members of Aeolidida. This hypothesis suggests that shifting to new prey items leads to an increase in niche availability, similar to the effect of habitat preference shifts in some groups [39]. This hypothesis is broader than the first, in that it refers to switches between prey types at higher levels of organization. The consequences of this type of switching relate to species that, following a switch, are able to prey on multiple taxa within a general prey type, rather than explicitly focusing on those that specialize on certain prey species. This pattern has been found in only a few taxa [34, 40, 41]. Diversification in this context relates more to the expansion of possible prey types rather than specialization.

Given the variety of prey type preferences exhibited by its members, Cladobranchia constitutes an excellent system to explore the relationship between prey shifting and cladogenesis. Until now, it has not been possible to test how prey choice has evolved through the history of this group, because existing cladobranch phylogenies are notoriously poorly resolved. Support for Cladobranchia as a monophyletic group is high [23, 42, 43], but the relationships among major lineages within Cladobranchia have long been problematic [22, 23, 42, 44–47]. However, a recent phylogenomic study provided evidence that these lineages could be resolved with RNA-Seq data [43]. In addition to the growing availability of RNA-Seq data for this group, the prey preferences of the majority of species within this group have been published (e.g., [15, 16, 18–20, 48]).

To address the role of dietary specialization and host shifts in the evolution of this group and resolve outstanding systematics issues, we reconstruct the phylogeny of Cladobranchia using RNA-Seq data. In this study, we increase the taxon sampling compared to previous phylogenomic work on Cladobranchia [43] by incorporating additional diversity from both previously sampled clades (Aeolidida and Dendronotida) and the previously unsampled Arminida (as defined in [23], though we only include members of Arminidae). In addition, we seek to address patterns of prey type switching among and within the major lineages of cladobranchs by assessing prey type preference for each taxon included in the phylogenetic analyses, and using these data to reconstruct the most likely ancestral prey type preference for each node in the tree. These analyses provide the means to examine the prevalence of prey type switching within Cladobranchia in order to provide a framework for studying how dietary preferences may have affected evolution within this group.

## Methods

### Organismal sampling

One or two specimens of each of 16 representative species were collected in tide pools or via snorkeling or SCUBA (self-contained underwater breathing apparatus; under AAUS certification) using a variety of methods including direct collection, substrate collection, and non-destructive collecting under rocks. A visual examination was used for confirmation of identity using field guides for the Caribbean [13] and the Indo-Pacific [14]. Barcode sequences and expert opinions were used when the identity of specimens was still uncertain. Images of select specimens are in Additional file 1: Figure S1 and Additional file 2: Figure S2. One of the two specimens was placed in RNAlater solution (Qiagen, Hilden, Germany) for RNA preservation and frozen at -80 ºC within one week of collection to prevent RNA degradation. Some specimens in RNAlater were instead stored at -20 ºC within 24 h and remained there for up to a month. A second specimen of each species, when available, was fixed as a voucher for morphological analysis, first in 10% formalin and subsequently preserved in 70% ethanol for long-term storage. Voucher specimens were deposited in the Smithsonian National Museum of Natural History (NMNH) and are available for study under the catalog numbers provided in Additional file 3: Table S5.

For *Bulbaeolidia alba*, *Hancockia uncinata*, *Unidentia angelvaldesi*, *Bornella anguilla*, *Dermatobranchus* sp., *Phestilla* sp., and *Eubranchus rustyus*, we were unable to obtain morphological vouchers. Cella et al. [49] proposed to use the genus name *Tenellia* for species previously assigned to *Catriona*, *Cuthona* and *Phestilla*, but recognized that the assignment of species to *Tenellia* is problematic due to the absence of morphological synapomorphies. Thus, we chose to temporarily maintain species in the genera *Catriona*, *Cuthona* and *Phestilla* until the additional studies suggested by Cella et al. [49] are carried out. We generated RNA-Seq data for 16 Cladobranchia species, downloaded data for 19 additional Cladobranchia species from the NCBI Sequence Read Archive (SRA) and obtained two RNA-Seq datasets from colleagues at Georgia State University. Three outgroup RNA-Seq datasets were also obtained from the SRA: two representatives of Anthobranchia (the sister taxon of Cladobranchia; [22, 46]), and one of Pleurobranchoidea (the sister taxon to Nudibranchia [50]). Specimen data, SRA numbers and barcode GenBank numbers are listed in Additional file 3: Table S5.

### RNA extraction and sequencing

A 20–100 mg tissue sample was taken from the anterior of each animal and homogenized using a motorized pestle. In some cases, the specimen was so small the entire animal was used. After homogenizing for 1–2 min the tissue was flash-frozen in liquid nitrogen for subsequent homogenizing until tissue mixture was fully uniform. 500 μL of TRIzol Reagent (Life Technologies, Carlsbad, CA, USA) was then added and the mixture was homogenized again. This procedure was repeated until the solution was deemed fully homogenized. Once this process was complete, an additional 500 μL of TRIzol Reagent was added to the solution and the mixture was left at room temperature for five min.

Following the five min incubation, 100 μL of 1-Bromo-3-chloropropane was added to the solution, which was subsequently mixed thoroughly. The mixture was then left at room temperature for five min, and then centrifuged at 16,000 g for 20 min at 8 ºC. The top aqueous phase was then removed and placed in another tube where 500 μL of 100% isopropanol was added, and stored overnight at -20 ºC for RNA precipitation.

After precipitation, the samples were centrifuged at 17,200 g for 10 min at 4 ºC. The supernatant was then removed and the pellet washed with freshly prepared 75% ethanol. The sample was then centrifuged at 7,500 g for 5 min at 4 ºC. The supernatant was removed and the pellet air-dried for 1 to 2 min (or until it looked slightly gelatinous and translucent). The total RNA was then re-suspended in 10–30 μL of Ambion Storage Solution (Life Technologies, Carlsbad, CA, USA), and 1 μL of SUPERase•In (Thermo Fisher Scientific, Waltham, Massachusetts, USA) was added to prevent degradation.

Total RNA samples were submitted to the DNA Sequencing Facility at University of Maryland Institute for Bioscience and Biotechnology Research, where quality assessment, library preparation, and sequencing were performed. RNA quality assessment was done with a Bioanalyzer 2100 (Agilent Technologies, Santa Clara, CA, USA), and samples with a concentration higher than 20 ng/μL were used for library construction. Library preparation used the Illumina TruSeq RNA Library Preparation Kit v2 (Illumina, San Diego, CA, USA) and 200 bp inserts; 100 bp, paired-end reads were sequenced with an Illumina HiSeq1000 (Illumina, San Diego, CA, USA).

### Quality control and assembly of reads

Reads that failed to pass the Illumina “Chastity” quality filter were excluded from our analyses. Reads passing the quality filter were assembled using Trinity (version 2.1.1; [51]) with default settings, which required assembled transcript fragments to be at least 200 bp in length.

### Orthology assignment

Translated transcript fragments were organized into orthologous groups corresponding to a custom gastropod-specific core-ortholog set of 3,854 protein models [43] using HaMStR (version 13.2.2; [52]), which in turn used FASTA (version 36.3.6d; [53]), GeneWise (version 2.2.0; [54]), and HMMER (version 3.1b2; [55]). In the first step of the HaMStR procedure, substrings of assembled transcript fragments (translated nucleotide sequences) that matched one of the gastropod protein models were provisionally assigned to that orthologous group. To reduce the number of highly divergent, potentially paralogous sequences returned by this search, we set the E-value cutoff defining an HMM hit to 1e-05 (the HaMStR default is 1.0), and retained only the top-scoring quartile of hits. In the second HaMStR step, the provisional hits from the HMM search were compared to the reference taxon, *Aplysia californica*, and retained only if they survived a reciprocal best BLAST hit test with the reference taxon using an E-value cutoff of 1e-05 (the HaMStR default was 10.0). In our implementation, we substituted FASTA [53] for BLAST [56] because FASTA programs readily accepted our custom amino acid substitution matrix (GASTRO50; [43]).

### Construction of data matrix and paralogy filtering

Protein sequences in each orthologous group were aligned using MAFFT (version 7.187; [57]). We used the --auto and --addfragments options of MAFFT to align transcript fragments to the *Aplysia californica* reference sequence, which was considered the existing alignment. We converted the protein alignments to corresponding nucleotide alignments using a custom Perl script. A maximum likelihood tree was inferred using GARLI (Genetic Algorithm for Rapid Likelihood Inference version 2.1; [58]) for each orthologous group where at least 75% of the taxa were present (716 orthologous groups), and was given as input to PhyloTreePruner (version 1.0; [59]). Orthologous groups that showed evidence of out-paralogs for any taxa (352 orthologous groups out of 716) were pruned according to the default PhyloTreePruner protocol, which removes all additional sequences outside of a maximally inclusive sub-tree. For orthologous groups containing in-paralogs, multiple sequences were combined into a single consensus sequence for each taxon, and orthologous groups for which fewer than 75% of taxa remained were discarded. This process left 406 orthologous groups eligible for inclusion in data matrices. Individual orthologous group alignments were concatenated (*nt123* matrix) (Table 1). Codons not represented by sequence data in at least four taxa were then removed (*nt123sitesremoved* matrix).

**Table 1:** Data matrix statistics for each of the two data matrices

Data matrix	# of nucleotide positions	% complete	% of ambiguous sites
*nt123*	966,888	33.1	0.08
*nt123sitesremoved*	605,934	50.7	0.13

### Phylogenetic analyses

Four separate phylogenetic analyses were completed in this study: (i) an analysis with the *nt123* data matrix partitioned by codon position (*nt123partitioned*) by assigning different model parameters and rates to the three types of codon positions, (ii) an analysis with the *nt123sitesremoved* data matrix partitioned by codon position (*nt123sitesremoved_partitioned*), (iii) an analysis of the *nt123* matrix partitioned by codon position, but excluding the third position (*nt12partitioned*), and (iv) an analysis of the unpartitioned *nt123sitesremoved* data matrix (*nt123sitesremoved_unpartitioned*). To conduct all four phylogenetic analyses we used GARLI (version 2.1; [58]) through the GARLI web service hosted at molecularevolution.org [60]. We used the default settings in GARLI, including a general time reversible substitution model (GTR; [61]) with a rate heterogeneity model with a proportion of invariant sites estimated (+I; [62]) and the remainder with a gamma distribution (+G; [63]), along with stepwise-addition starting trees. Post-processing of the phylogenetic inference results was performed by the GARLI web service at molecularevolution.org using DendroPy [64] and the R system for statistical computing [65]. For all analyses, 1000 bootstrap replicates were generated and a best tree search was performed with 10 search replicates.

### Ancestral state reconstruction

We conducted a literature search to collect prey preference data for all nudibranch taxa in our phylogeny and coded each species as Anthozoa: Octocorallia, Anthozoa: Hexacorallia, Hydrozoa, Scyphozoa, Bryozoa, Crustacea, Gastropoda eggs, or generalist, for a total of eight states (Additional file 4: Table S4). In the cases where more than one type of prey is fed upon by an individual species, we provide that information and run additional analyses to test the effect of these alternatives on the final results. The final analysis incorporates the prey type for each species that that species has been observed to feed on more than 50% of the time. Data was compiled primarily from review papers on feeding and defense in nudibranchs [15, 16, 18–20], field guides [13, 66], one additional paper [67], and web sources where necessary [68–71]. Though limited, the taxon selection in this study represents a large portion of the morphological and ecological diversity of Cladobranchia, including the diversity of prey type preferences. Using these character states, we compared the fit of three discrete trait models using the AICcmodavg 2.0-4 package [72] in R 3.3.1 [65]. We assessed fit for models where: (i) all transition rates were equal (ER); (ii) forward and reverse transitions were equal between states (i.e. symmetrical, SYM); and (iii) all transition rates were different (ARD) using the corrected Akaike information criterion (AICc). The ER model (AICc = 100.61) was a better fit to the data than either the SYM model (AICc = 118.53) or the ARD model (AICc = 165.16). The final ancestral state reconstruction analysis was completed using the ace function, in the APE package [73], under the ER model using default parameters and a joint reconstruction approach. The ace function uses a Markov model employing a maximum likelihood approach. In this analysis, the reconstructed ancestral states that are returned are given as the proportion of the total likelihood calculated for each state for each node.

## Results

### Assembly and data matrix properties

The raw number of RNA-Seq reads for each species ranged from 25,756,442 to 133,156,930 (x̅ ≈ 49M reads; Additional file 5: Table S1). Once assembled, the number of transcript fragments per sample ranged from 71,967 to 295,127 (x̅ = 146,403; Additional file 6: Table S2). N50 ranged from 395 to 1,058 bp (x̅ = 716 bp). The transcript fragments from each assembly that matched the HaMStR database ranged from 615 to 2,013 (x̅ = 1,282). However, the number of matches to unique orthologous groups ranged from 512 to 1,198 (x̅ = 935). The mean length of transcript fragment matches to the HaMStR database was 282 amino acids. HaMStR results are presented in Additional file 7: Table S3.

### Phylogenetic results

**Fig. 2:**
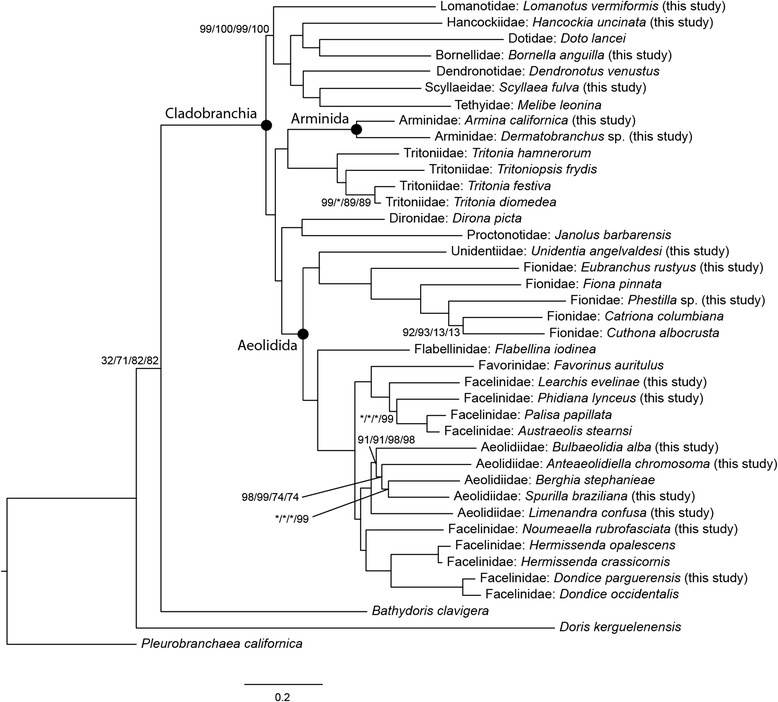
The maximum likelihood topology from the *nt123_partitioned* analysis, with bootstrap support values from each analysis labeled on some nodes (*nt123_partitioned/nt123sitesremoved_partitioned/nt123sitesremoved_ unpartitioned/nt12partitioned*). All unlabeled nodes have 100% bootstrap support in all analyses

Results from all analyses supported Cladobranchia as a monophyletic group with a bootstrap (BS) value of 100% (Fig. 2). Arminidae (Arminida) is also supported as monophyletic (BS = 100%), and is sister (BS = 100%) to Tritoniidae (BS = 100%).

Dendronotida is non-monophyletic across all topologies, with dendronotid taxa comprising two major clades. The first of these clades is sister to all other cladobranchs (BS = 100%) and contains *Doto* (Dotidae), *Bornella* (Bornellidae), *Hancockia* (Hancockiidae), *Scyllaea* (Scyllaeidae), *Melibe* (Tethyidae), *Dendronotus* (Dendronotidae), and *Lomanotus* (Lomanotidae).

Aeolidida is supported as monophyletic (BS = 100%) across all topologies, containing *Flabellina* (Flabellinidae), *Berghia*, *Spurilla*, *Bulbaeolidia*, *Anteaeolidiella,* and *Limenandra* (Aeolidiidae)*, Hermissenda*, *Dondice*, *Noumeaella*, *Favorinus*, *Palisa*, *Austraeolis*, *Learchis,* and *Phidiana* (Facelinidae)*, Fiona, Cuthona*, *Catriona, Phestilla*, and *Eubranchus* (Fionidae), and *Unidentia* (Unidentiidae). All families within Aeolidida where multiple taxa from the same family are included are supported as monophyletic, with the exception of Facelinidae, which is polyphyletic and forms two separate clades.

Two taxa previously unassigned to any of the three major clades, *Dirona* (Dironidae) and *Janolus* (Proctonotidae), are supported as sister taxa (BS = 100%) and form a clade that is sister to Aeolidida (BS = 100%).

### Ancestral state reconstruction analysis

**Fig. 3:**
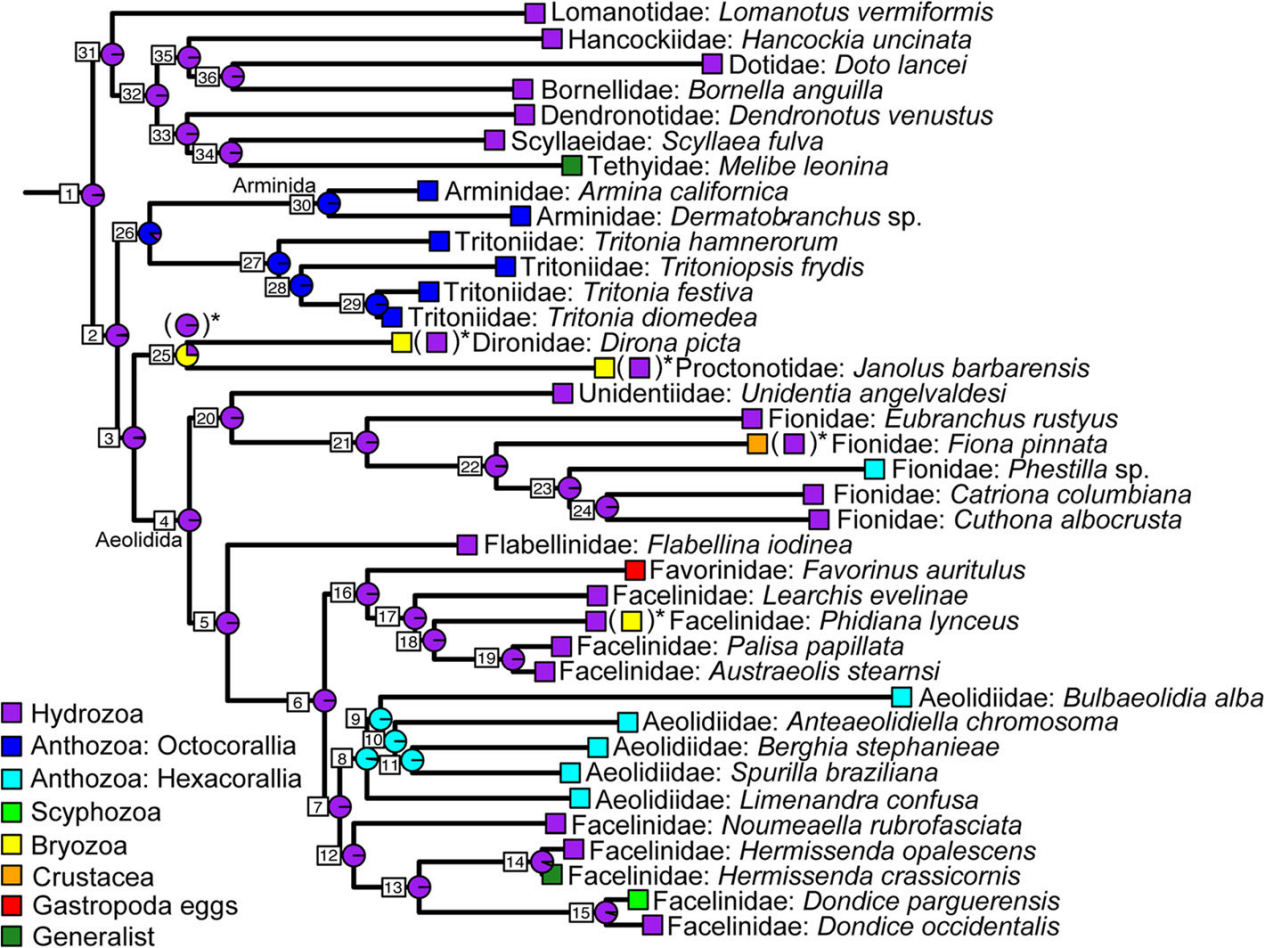
Ancestral state reconstruction results for the evolution of diet preference in Cladobranchia. Pie charts on the nodes are scaled likelihoods calculated using the ace function in APE. Alternative states and results are indicated in parentheses with an asterisk at the tips of the tree and nodes, and only alternative node states with greater than or equal to 5% difference from the original reconstruction are shown. Nodes are also labeled with numbers consistent with Table 2

The ancestral state reconstruction results support the hypothesis that the most recent common ancestor (MRCA) of Cladobranchia preyed upon species of Hydrozoa (Fig. 3). A cladobranch that preyed upon Hydrozoa also appears to be the MRCA for Aeolidida and the clade composed of most of the taxa assigned to Dendronotida, as well as the rest of the MRCAs along the backbone of the tree. However, a taxon that preyed upon Octocorallia species is the likely MRCA for the Arminidae + Tritoniidae clade (88.78% of the scaled likelihood), a taxon that preyed upon bryozoans or hydrozoans is the most likely MRCA for the *Dirona* + *Janolus* clade (74.44% of the scaled likelihood; Fig. 3, Table 2), and the MRCA for Aeolidiidae most likely fed upon species within Hexacorallia (98.03% of the scaled likelihood). Additional ancestral state reconstruction analyses were completed to evaluate the effects of alternative prey types for certain taxa on the overall reconstruction of ancestral states (Additional file 8: Table S6, Additional file 9: Table S7, Additional file 10: Table S8, Additional file 11: Table S9, Additional file 12: Table S10). With the exception of the ancestral node of the *Dirona* + *Janolus* clade, which changes to >97% of the scaled likelihood supporting a Hydrozoa feeding ancestor in three of the alternative analyses, the results are robust to these changes. The scaled likelihoods across all other nodes within each of the alternative analyses remain within 5% of the value in the original analysis.

**Table 2:** Ancestral state reconstruction results for the evolution of diet preference in Cladobranchia. This table provides the percentage (%) of the total likelihood assigned to each state for each node. The node numbers correspond to those provided in Fig. 3

Node	Octocorallia	Hexacorallia	Hydrozoa	Bryozoa	Scyphozoa	Crustacea	Gastropoda eggs	Generalist
1	0.8141	0.0100	98.8615	0.2744	0.0100	0.0100	0.0100	0.0100
2	1.6933	0.0096	97.6957	0.5631	0.0096	0.0096	0.0096	0.0096
3	1.1047	0.0113	97.8230	1.0160	0.0113	0.0113	0.0113	0.0113
4	0.0069	0.0003	99.9855	0.0064	0.0002	0.0003	0.0002	0.0002
5	0.0010	0.0007	99.9957	0.0010	0.0004	0.0004	0.0004	0.0004
6	0.0002	0.0163	99.9808	0.0002	0.0003	0.0002	0.0018	0.0004
7	0.0007	0.1722	99.8198	0.0007	0.0017	0.0007	0.0014	0.0027
8	0.0080	98.0323	1.9194	0.0080	0.0081	0.0080	0.0080	0.0081
9	0.0013	99.7978	0.1946	0.0013	0.0013	0.0013	0.0013	0.0013
10	0.0001	99.9881	0.0111	0.0001	0.0001	0.0001	0.0001	0.0001
11	0.0002	99.9958	0.0028	0.0002	0.0002	0.0002	0.0002	0.0002
12	0.0004	0.0418	99.9485	0.0004	0.0026	0.0004	0.0006	0.0051
13	0.0071	0.0202	99.5668	0.0071	0.1256	0.0071	0.0071	0.2590
14	0.0095	0.0103	93.6216	0.0095	0.0159	0.0095	0.0095	6.3140
15	0.0225	0.0236	95.2642	0.0225	4.5788	0.0225	0.0225	0.0434
16	0.0051	0.0128	99.8505	0.0051	0.0051	0.0051	0.1112	0.0052
17	0.0002	0.0003	99.9974	0.0002	0.0002	0.0002	0.0015	0.0002
18	0.0001	0.0001	99.9991	0.0001	0.0001	0.0001	0.0003	0.0001
19	0.0000	0.0000	99.9997	0.0000	0.0000	0.0000	0.0000	0.0000
20	0.0023	0.0014	99.9885	0.0022	0.0011	0.0022	0.0011	0.0011
21	0.0086	0.0269	99.8573	0.0086	0.0084	0.0735	0.0084	0.0084
22	0.0452	0.3201	98.4388	0.0452	0.0452	1.0152	0.0452	0.0452
23	0.0284	0.5362	98.9631	0.0284	0.0284	0.3586	0.0284	0.0284
24	0.0066	0.0935	99.8105	0.0066	0.0066	0.0631	0.0066	0.0066
**25**	**0.4701**	**0.2032**	**24.0771**	**74.4369**	**0.2032**	**0.2032**	**0.2032**	**0.2032**
**25** ^**alt1**^	**0.0049**	**0.0021**	**99.9846**	**0.0021**	**0.0021**	**0.0000**	**0.0021**	**0.0021**
**25** ^**alt2**^	**0.0664**	**0.0287**	**99.4038**	**0.3865**	**0.0287**	**0.0287**	**0.0287**	**0.0287**
**25** ^**alt3**^	**0.1369**	**0.0585**	**97.8795**	**1.6908**	**0.0585**	**0.0587**	**0.0585**	**0.0585**
26	88.7837	0.0555	10.8222	0.1165	0.0555	0.0555	0.0555	0.0555
27	99.9377	0.0016	0.0525	0.0019	0.0016	0.0016	0.0016	0.0016
28	99.9924	0.0003	0.0056	0.0004	0.0003	0.0003	0.0003	0.0003
29	99.9998	0.0000	0.0000	0.0000	0.0000	0.0000	0.0000	0.0000
30	99.7727	0.0076	0.1805	0.0086	0.0076	0.0076	0.0076	0.0076
31	0.1248	0.0022	99.8216	0.0425	0.0022	0.0022	0.0022	0.0023
32	0.0009	0.0001	99.9982	0.0003	0.0001	0.0001	0.0001	0.0003
33	0.0020	0.0016	99.9689	0.0017	0.0016	0.0016	0.0016	0.0210
34	0.0316	0.0314	99.2906	0.0314	0.0314	0.0314	0.0314	0.5209
35	0.0005	0.0004	99.9972	0.0004	0.0004	0.0004	0.0004	0.0004
36	0.0032	0.0032	99.9776	0.0032	0.0032	0.0032	0.0032	0.0032

Bold values are those on nodes different by greater than or equal to 5% in at least one alternative analysis. Abbreviations: Alt1, analysis using all alternative states; Alt2, analysis using the alternative state for *Dirona picta*; Alt3, analysis using the alternative state for *Janolus barbarensis*

## Discussion

In this study we significantly increased the breadth of RNA-Seq sampling in Cladobranchia in order to generate a robust phylogenetic hypothesis, and provide a framework for the evolution of prey type preference within this group.

### Prey preference evolution in Cladobranchia

Well-supported clades recovered within Cladobranchia appear to be strongly associated with prey groups. Most of the larger clades recovered in the phylogenetic tree prey almost exclusively on particular types of organisms (Fig. 3), such as Aeolidiidae on Hexacorallia, Arminida + Tritoniidae on Octocorallia, and multiple clades that prey on Hydrozoa. This result is in opposition to previous studies [22, 45], which indicated that groupings within Cladobranchia contained taxa that fed on a broad range of prey types. The results here support the idea that prey preference within Cladobranchia may be a taxonomically useful trait for placing taxa into some groups. Past taxonomic work on Cladobranchia has focused on different anatomical features to diagnose groups, such as the presence of rhinophoral sheaths (Dendronotida) or oral veils (Arminida) [22]. In the future, incorporating feeding adaptations to particular prey taxa may accelerate taxonomic progress in the group.

Our results indicate that prey preference shifts from one major taxon to another are relatively rare in Cladobranchia. The ancestral state reconstruction unambiguously supports an ancestor for Cladobranchia that preyed upon Hydrozoa (Fig. 3). This analysis also suggests at least five transitions from hydrozoan to other prey taxa, such as Hexacorallia (Anthozoa), Octocorallia (Anthozoa), and Scyphozoa. Interestingly, the clade containing *Dirona* and *Janolus* has expanded to feeding on Bryozoa in addition to Hydrozoa, rather than shifting to Bryozoa exclusively. This expansion is mirrored in *Phidiana*, which is also able to feed on members of both taxa. Overall, expansions to feeding on multiple types of prey have occurred at least six times in Cladobranchia, leading to multiple generalist taxa (*Melibe* and *Hermissenda*) and those that can feed on both Hydrozoa and either Bryozoa or Crustacea. These are cases in which diversification might be related more to an increase in options rather than specialization.

The mechanism by which the evolution of prey preference is constrained is unknown, but it could result from relative difficulty in evolving specific traits for protection against nematocysts (or other defenses) from various cnidarian prey groups. Although it is possible for some species (e.g., *Phidiana hiltoni*) to prey upon different cnidarian species [74], there are few examples of cladobranchs preying on multiple, taxonomically distant cnidarians (only *H. crassicornis* in this study). Cladobranchs require a series of adaptations to prevent cnidarian nematocysts from firing or minimizing the damage in the case of firing [75], including mucous secretions. These secretions appear to be specific to the prey species in one studied case [76], and may be why switching between types of prey is much more challenging and occurs much less frequently than previously thought. Species of Cladobranchia that do not prey on cnidarians, such as *Favorinus*, can easily switch between egg masses of distantly related gastropods, including aplysiids, sacoglossans, and other nudibranchs [48], lending support to this hypothesis.

Previous work has suggested that dietary specialization on particular prey types was crucial in the evolution of Euthyneura [16, 17, 77], and has been proposed as a “driving force” in heterobranch sea slug evolution [38, 78]. Dietary specialization has also been considered a contributing factor in the species richness of Nudibranchia as a whole [17], and especially cladobranchs [16, 18] in conjunction with the evolution of nematocyst sequestration. This hypothesis is entirely plausible when looking at the numbers of species in prey groups and how those correlate with species diversity in the cladobranch predators. Although Bryozoa has nearly 6,000 species [79], there are fewer than 50 species within Cladobranchia that prey on members of this group [48]. Conversely, more than 700 species of cladobranchs prey on Hydrozoa, a clade of cnidarians with ~3,500 species [79]. This drastic difference is primarily due to Aeolidida, which contains a large proportion of the taxa that prey on Hydrozoa, and which appears to have diversified primarily while preying on hydrozoans. Our results do not support the hypothesis that prey type shifts lead to morphological adaptations that increase diversity, as the distinct radular morphology found in Aeolidida is not associated with a prey type shift according to our ancestral state reconstruction. However, Aeolidida is also one of two lineages where nematocyst sequestration is known to have evolved within Cladobranchia [80]. Given that hydrozoans are known to have the highest diversity of nematocyst types [81], the ability to sequester nematocysts may have had an impact on diversification.

The hypothesis that the diversity of larger clades within Cladobranchia is related to the frequency of major prey type shifts is not supported by these results. Instead, we suspect that if shifts in diversification associated with diet in Cladobranchia are going to be found, these may occur within groups that prefer a major prey type (e.g., at the family or genus level), where more species-specific prey shifting is likely to occur [16, 24]. The literature indicates that in groups where taxa are specialized on particular prey species, shifting to a new prey (or host) species often leads to speciation and diversification [82–84]. This pattern is found in many metazoan taxa, including flies [85], amphipods [35, 86, 87], alpheid shrimp [88, 89], barnacles [36], whelks [90], gobies [37], and sacoglossan gastropods [91, 92], and has been extensively investigated in phytophagous insects (reviewed in [93]). Within Nudibranchia, a large subset of taxa exhibit specialization on a single species, with many others preferring only two or three prey species [94]. We suspect that in the case of Cladobranchia, this specialization and prey shifting at the species level may be the primary impact that prey preference has on the diversification rate across lineages, rather than shifts to new prey types, as is true in many herbivorous insect lineages (reviewed in [93]). Tests of this hypothesis require a broader sampling of members of Cladobranchia for both the phylogenetic inference and species-specific prey preference data.

### Systematics of Cladobranchia and prey preference within individual clades

Based on the phylogenetic hypothesis presented here, the monophyly of Cladobranchia is reinforced with full bootstrap support across all analyses. Though monophyly was indicated in previous morphological [22] and molecular [42–46, 95] analyses, there has also been a study suggesting paraphyly [23], though the authors of that paper contended that this might be due to a deletion of a string of nucleotides within one lineage (*Melibe*) that was biasing the results.

### Arminida

The most significant systematics results from this study involve Arminida, a group not included in the one previous phylogenomic study of Cladobranchia [43]. Arminida, when first described, comprised the genera *Janolus* and *Dirona*, among other taxa, including Arminidae [96]. The inclusion of *Janolus* and *Dirona* within Arminida renders this group paraphyletic in both morphological and molecular analyses [22, 97], and they, along with others (Charcotiidae and Pinufiidae), have since been removed from Arminida and are considered unassigned members of Cladobranchia [22]. The analyses presented here support this exclusion of *Janolus* and *Dirona* from Arminida, consistent with recent studies [22, 42, 98]. Both of these genera primarily prefer bryozoan prey, but also feed on members of Hydrozoa.

There is strong support for Arminidae (one of two families within Arminida) as the sister group to Tritoniidae, which is a novel result. This result is in agreement with only one previous phylogenetic hypothesis, which was generated using 18S rDNA data [42]. In all other previous studies, taxa from Arminida had been either unplaced within the Cladobranchia phylogeny [46, 95, 98] or supported as sister to various other combinations of taxa from Dendronotida and Aeolidida [42]. The position of Tritoniidae in the previous phylogenomics study of Cladobranchia was uncertain [43]. It appears that prey preference is particularly relevant for the evolution of Tritoniidae + Arminidae as species within this group prey exclusively on Octocorallia. Species within Octocorallia are known for their noxious chemical defenses in addition to the nematocysts present in their tissues [99, 100], and these defenses could help explain why a switch to octocorals has occurred rarely within Cladobranchia.

A caveat does exist, however, in regard to the classification of these clades. Both of the taxa from Arminida included within the present analyses (*Armina* and *Dermatobranchus*) are members of the family Arminidae; thus, the monophyly of Arminida (containing Arminidae and Doridomorphidae) as a whole has not yet been rigorously tested. That said, the un-sampled family within Arminida, Doridomorphidae, is monotypic and its sole species lives on the blue coral *Heliopora coerulea*, an octocoral with a massive calcium carbonate skeleton [14, 101]. This is congruent with the dietary evolution results offered here.

### “Dendronotida”

With regard to Dendronotida, the analyses presented here strongly contradict monophyly. The majority of families within Dendronotida (Lomanotidae, Hancockiidae, Dotidae, Bornellidae, Scyllaeidae, Tethyidae, and Dendronotidae) form a single clade that is sister to all other species within Cladobranchia. This clade and the relationships within it are fully supported (BS = 100%) by all analyses presented here. Lomanotidae as sister to the rest of the species within this clade is a result novel to this study, with most previous morphological and molecular analyses [23, 46] supporting alternative topologies, though support was mostly low for these hypotheses.

The rest of the group contains two clades, the first of which is one where Bornellidae is sister to Dotidae and Hancockiidae is sister to the Dotidae + Bornellidae assemblage. This result is also novel as compared to most previous studies [46, 98, 102–105]. In morphological analyses in particular, both *Hancockia* and *Doto* have been considered “problematic” genera [22], and as such have mostly been unplaced (*Hancockia*) [22] or unassigned (*Doto*) [47] within the Cladobranchia phylogeny. The analyses presented here, however, very strongly support the position of these genera in the tree, and therefore provide a much stronger hypothesis for their relationships. The second clade within this grouping contains sister groups Scyllaeidae and Tethyidae, as well as Dendronotidae, which is sister to the Scyllaeidae + Tethyidae assemblage. These relationships are consistent with most previous studies [23, 43, 46, 103], but similar to the Dotidae + Bornellidae + Hancockiidae clade, in other cases Scyllaeidae has been previously supported as sister to Dendronotidae, with Tethyidae (usually *Melibe* specifically) as an early branching lineage [98, 102, 106].

This clade containing the members of “Dendronotida” appears to be almost exclusively composed of taxa that prey on hydrozoans, with the exception of *Melibe*, which prefers crustaceans that it catches with a remarkable oral hood [107]. Species within Tritoniidae (originally assigned to Dendronotida) form a separate monophyletic group in all analyses as sister to Arminida, as discussed above. In addition, this topology supports the hypothesis that nematocyst sequestration evolved at least twice, because the genus *Hancockia* (the only non-aeolid genus to sequester nematocysts; Martin *et al.* 2009) does not form a clade with Aeolidida.

### Aeolidida

Aeolidida is fully supported as monophyletic, consistent with previous studies [22, 42, 43, 45]. The first of two clades within Aeolidida is made up of taxa from Facelinidae, Aeolidiidae and Flabellinidae. The family Facelinidae forms two separate clades within this group, while Aeolidiidae is monophyletic. The relationships within this clade are consistent with most previous studies using molecular data [43, 46, 98, 108–111]. Based on these results, Facelinidae should likely be split into two separate families, with one clade retaining the name Facelinidae and the other assigned a more appropriate identifier. However, until a member of the genus *Facelina* (the type genus for this family) is included in the analyses (ideally the type taxon *Facelina auriculata*), it is impossible to say which clade should receive the Facelinidae designation. These results also include support for Aeolidiidae as a monophyletic group, at the base of which is one of two shifts to Hexacorallia prey within Aeolidida. The other shift occurs within the family Fionidae.

The second clade within Aeolidida is fully supported across all analyses (BS = 100%), and contains taxa from two families (Fionidae and Unidentiidae) [49]. The relationships between these taxa are also fully supported across all analyses, with the exception of the relationships within Fionidae (though the family itself is monophyletic with full support). Sister to the Fionidae is Unidentiidae. This position for Unidentiidae is a novel result. Only one study previously addressed the phylogenetic position of this family, using morphological data, and in that case Unidentiidae was found to be more closely related to members of Flabellinidae, Piseinotecidae, and Babakinidae [67]. This Fionidae + Unidentiidae clade in particular has multiple shifts to different prey types, including shifts to Crustacea (*Fiona*) and Hexacorallia (*Phestilla*).

## Conclusions

RNA-Seq data have recovered a well-supported phylogeny for Cladobranchia. The results of this study include a robust hypothesis of relationships between the major cladobranch clades, and indicate that some taxonomically diverse groups, such Dendronotida and Facelinidae, are not monophyletic. The ancestral state reconstruction indicates a strong phylogenetic correlation with prey preference within this group, indicating that host shifts are much more infrequent than previously thought. The mechanism causing evolution of prey preference to be constrained remains unknown, but it could result from difficulties in evolving specific traits for protection against nematocysts from various cnidarian prey groups and chemical compounds from Octocorallia. Future research of Cladobranchia would benefit from combined analyses of prey specialization and prey switching, nematocyst sequestration evolution, and diversification using broader sample coverage. The present study provides a framework for understanding major evolutionary trends in Cladobranchia and indicates that prey type specialization within this group has phylogenetic inertia.

## Additional files

Additional file 1: Figure S1. Select photographs of dendronotid and unassigned taxa used in this project, including: A) *Scyllaea fulva* (SRR3726701), B) *Dermatobranchus* sp. (SRR3726698; Photo credit: Karen Cheney), C) *Lomanotus vermiformis* (SRR3726706) and D) *Hancockia uncinata* (Photo credit: David Fenwick III). (TIFF 19274 kb)

Additional file 2: Figure S2. Select photographs of aeolid taxa used in this project, including: A) *Phidiana lynceus*, B) *Eubranchus rustyus* (SRR3726692; Photo credit: Craig Hoover), C) *Learchis evelinae* (SRR3726693), and D) *Spurilla braziliana*. (TIFF 14185 kb)

Additional file 3: Table S5. List of specimens examined in this study, including species name, locality, and morphological and molecular tissue voucher and barcode information. Sequence Read Archive accession numbers are also provided for each RNA-Seq dataset. (XLSX 41 kb)

Additional file 4: Table S4. Prey preference data used for the ancestral state reconstruction. (XLSX 48 kb)

Additional file 5: Table S1. Table of sequence read information for each sample. (XLSX 55 kb)

Additional file 6: Table S2. Trinity-assembly details, including number of transcript fragments and total number of bases assembled, as well as N50 and L50 statistics for each transcriptome. (XLSX 42 kb)

Additional file 7: Table S3. HaMStR statistics for each RNA-Seq dataset. (XLSX 43 kb)

Additional file 8: Table S6. Ancestral state reconstruction results for the evolution of diet preference in Cladobranchia with the alternative prey type states. (XLSX 51 kb)

Additional file 9: Table S7. Ancestral state reconstruction results for the evolution of diet preference in Cladobranchia with the alternative prey type state for *Dirona picta*. (XLSX 54 kb)

Additional file 10: Table S8. Ancestral state reconstruction results for the evolution of diet preference in Cladobranchia with the alternative prey type state for *Fiona pinnata*. (XLSX 54 kb)

Additional file 11: Table S9. Ancestral state reconstruction results for the evolution of diet preference in Cladobranchia with the alternative prey type state for *Janolus barbarensis*. (XLSX 54 kb)

Additional file 12: Table S10. Ancestral state reconstruction results for the evolution of diet preference in Cladobranchia with the alternative prey type state for *Phidiana lynceus*. (XLSX 54 kb)
