# Supplementary figures and images for: Prey preference follows phylogeny: evolutionary dietary patterns within the marine gastropod group Cladobranchia (Gastropoda: Heterobranchia: Nudibranchia)
Source: BMC Evol Biol. 2017 Oct 26;17:221. doi: 10.1186/s12862-017-1066-0 (PMC5659023; doi:10.1186/s12862-017-1066-0)

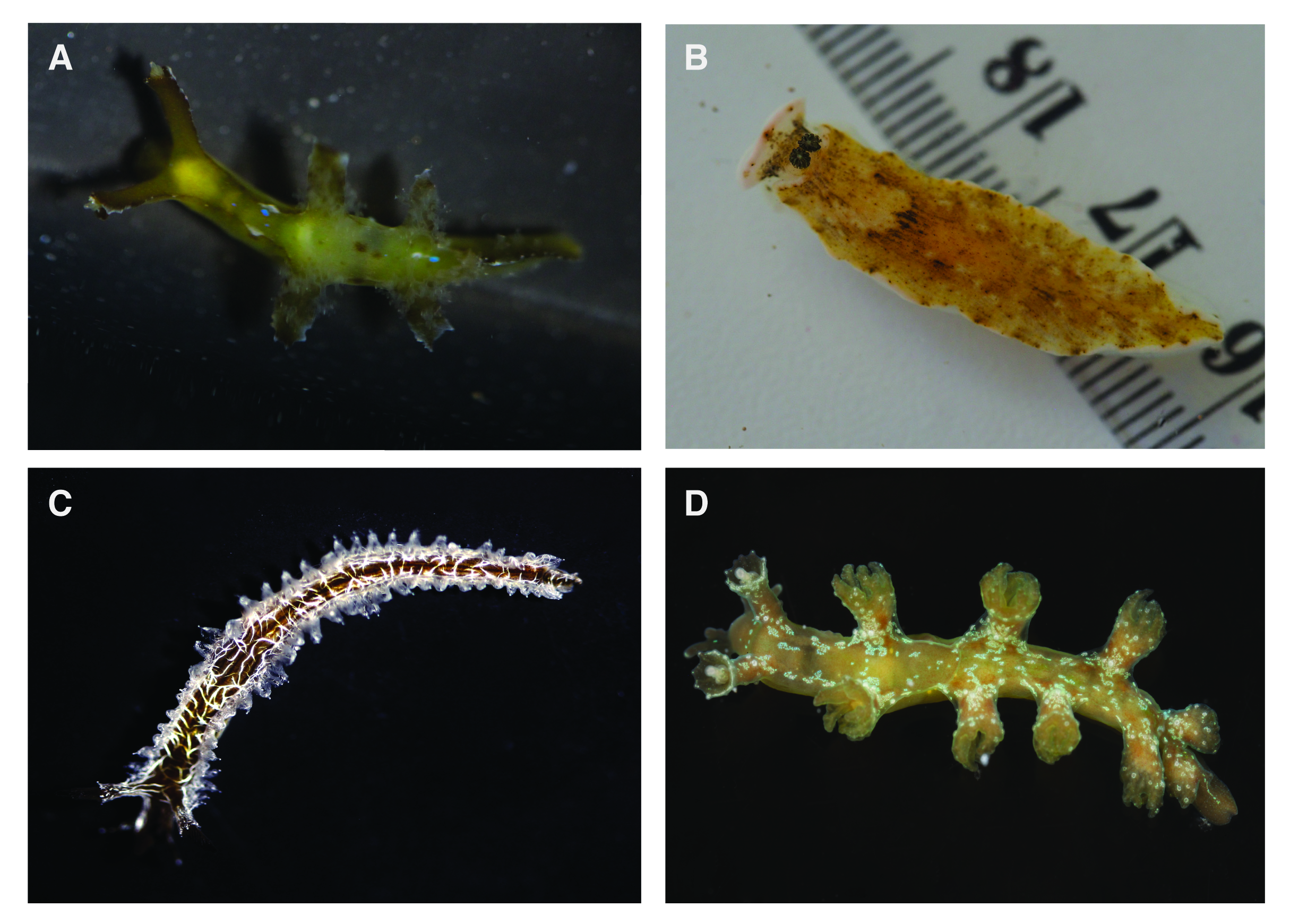

Supplement: Supplementary file 1 — Select photographs of dendronotid and unassigned taxa used in this project, including: A) Scyllaea fulva (SRR3726701), B) Dermatobranchus sp. (SRR3726698; Photo credit: Karen Cheney), C) Lomanotus vermiformis (SRR3726706) and D) Hancockia uncinata (Photo credit: David Fenwick III). (TIFF 19274 kb) [file 12862_2017_1066_MOESM1_ESM.tif]

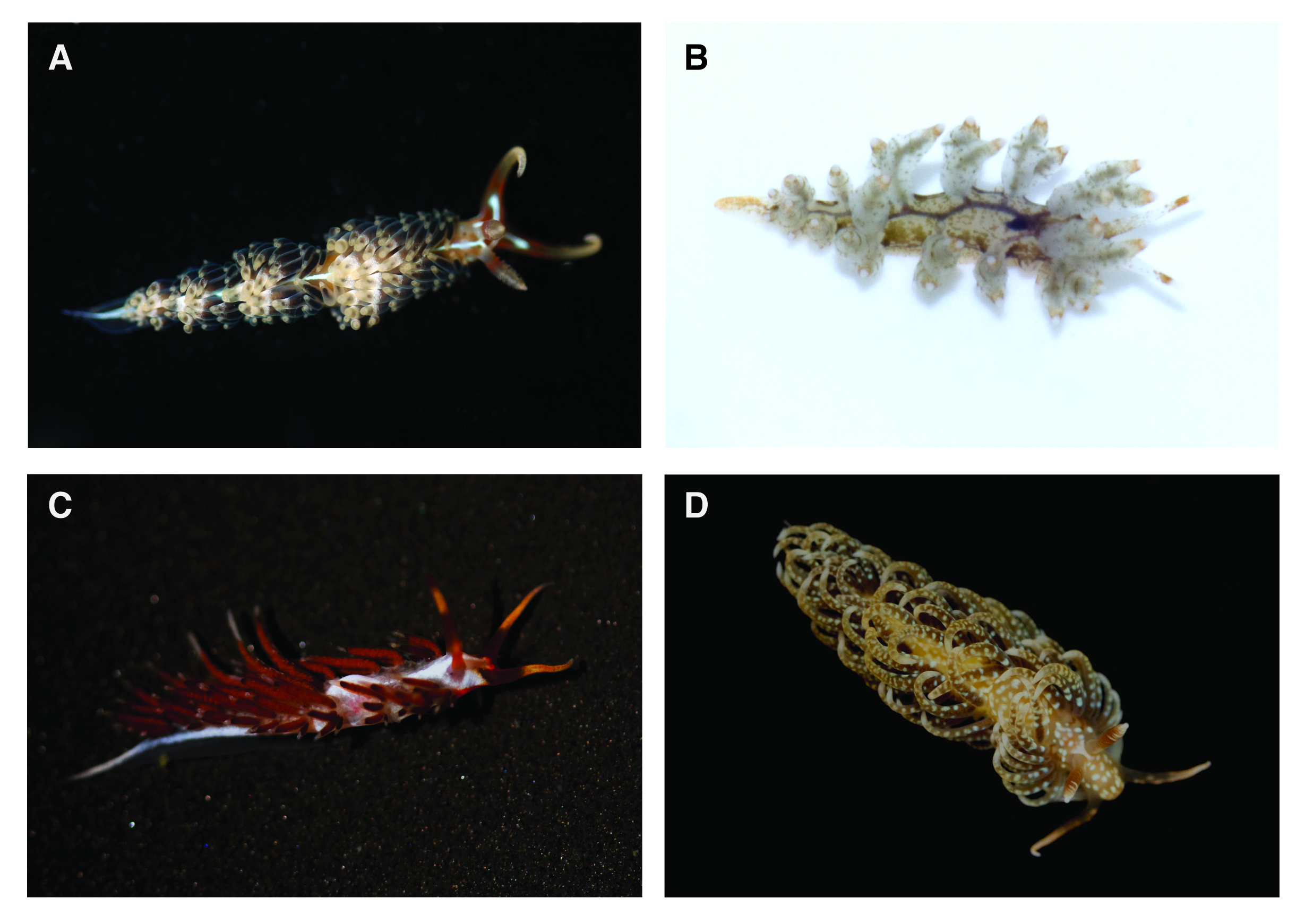

Supplement: Supplementary file 2 — Select photographs of aeolid taxa used in this project, including: A) Phidiana lynceus, B) Eubranchus rustyus (SRR3726692; Photo credit: Craig Hoover), C) Learchis evelinae (SRR3726693), and D) Spurilla braziliana. (TIFF 14185 kb) [file 12862_2017_1066_MOESM2_ESM.tif]
